# Chronic Hepatitis with Liver Granulomas in a Patient with Granuloma Annulare: A Case Report and Review of the Literature

**DOI:** 10.1155/2017/8768529

**Published:** 2017-02-23

**Authors:** Majid Alsahafi, Mohammed I. AlJasser, Sunil Kalia, H. M. Yang, Alnoor Ramji

**Affiliations:** ^1^Division of Gastroenterology, University of British Columbia, Vancouver, BC, Canada; ^2^Department of Medicine, King Abdulaziz University, Jeddah, Saudi Arabia; ^3^Department of Dermatology and Skin Science, University of British Columbia, Vancouver, BC, Canada; ^4^Department of Pathology and Laboratory Medicine, University of British Columbia, Vancouver, BC, Canada

## Abstract

Granuloma annulare (GA) is a benign granulomatous skin disorder of unknown etiology. GA is rarely associated with liver diseases. We report a unique case of chronic hepatitis with liver granulomas in a patient with GA. Despite an extensive workup, no clear etiology for the hepatitis was found. Based on the possible immune pathophysiology of GA and the presence of liver granulomas, the patient was treated with prednisone and azathioprine which resulted in complete normalization of the liver enzymes and concurrent improvement of GA. The association between liver diseases and GA is reviewed.

## 1. Introduction

Granuloma annulare (GA) is a benign cutaneous granulomatous disorder of unknown etiology. It often presents as annular, red-brown grouped papules which are often asymptomatic or only slightly pruritic. There are four clinical variants of GA: localized, generalized, perforating, and subcutaneous [1]. The pathogenesis of GA remains poorly understood but an immune mediated mechanism has been speculated [2, 3]. GA has been reported in association with several systemic disorders but a causative relationship has not been established [1].

GA is rarely reported in association with liver diseases. Here we report a unique case of a patient with GA, who presented with elevated liver enzymes and a biopsy revealing hepatic granulomas. To our knowledge, a similar association has not been previously reported.

## 2. Case Presentation

**Figure 1:**
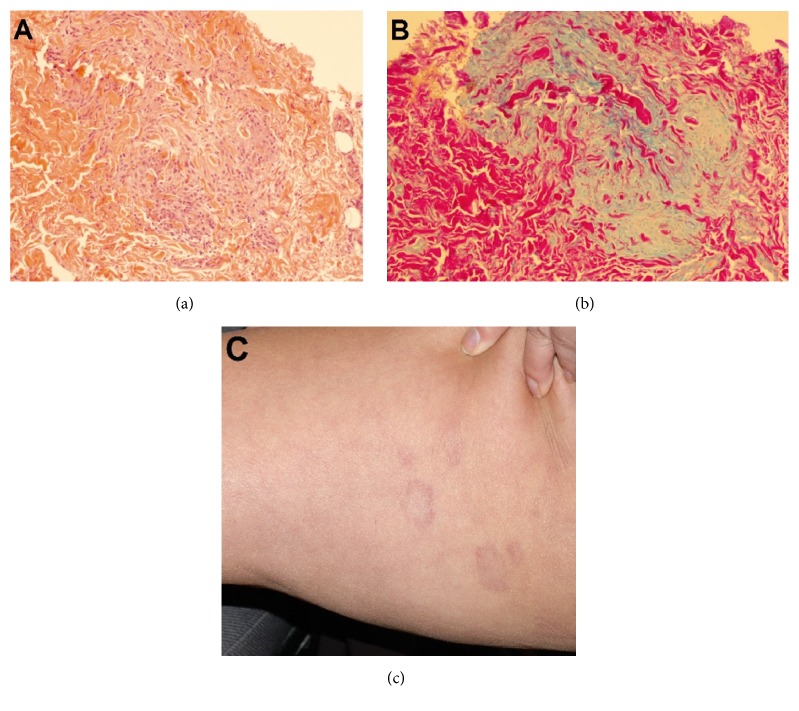
Granuloma annulare. (a) A palisading granuloma with central collagen degeneration (H&E stain, magnification ×100); (b) Alcian blue stain showing increased mucin deposition within the granuloma (Alcian blue stain, magnification ×100); (c) several annular red-brown plaques involving the thigh.

A 57 year-old woman was evaluated for elevated liver enzymes discovered on routine blood work on July 2010. Her blood work showed an alanine transaminase (ALT) at 177 (normal range 15–55 U/L), aspartate aminotransferase (AST) at 86 (normal range 15–45 U/L), and* alkaline phosphatase* (ALP) at 114 (normal range 30–105 U/L) with a normal gamma-glutamyl transferase (GGT) at 44 (normal range < 50 U/L). The patient was asymptomatic. She had no risk factors for viral hepatitis. She drinks alcohol only socially, considered minimal overall. She does not take any medications, including no over-the-counter drugs or herbal supplements. Her past medical history was significant for a 9-year history of generalized GA proven on two skin biopsies for which she receives phototherapy 3 times per week (Figures 1(a) and 1(b)). She has no family history of liver disease. Physical examination was unremarkable except for multiple generalized red-brown annular plaques involving the trunk and extremities (Figure 1(c)).

Laboratory investigations revealed normal complete blood count, renal profile, and* erythrocyte sedimentation rate*. Liver synthetic function tests including international normalized ratio (INR), bilirubin, and albumin were initially normal. Serology for hepatitis C, hepatitis B, cytomegalovirus, and* Epstein-Barr virus* were negative. Workup for autoimmune hepatitis revealed negative* antinuclear*,* anti-smooth-muscle*,* anti-liver kidney microsomal*, and* anti-mitochondrial antibodies*. Serum immunoglobulins levels were within the normal limits. Tissue transglutaminase antibodies test was negative. Serum ceruloplasmin and alpha-1-antitrypsin levels were normal. Transferrin saturation was not elevated. Angiotensin converting enzyme was slightly decreased. Abdominal ultrasound revealed a normal size liver with a slight coarse appearance.

**Figure 2:**
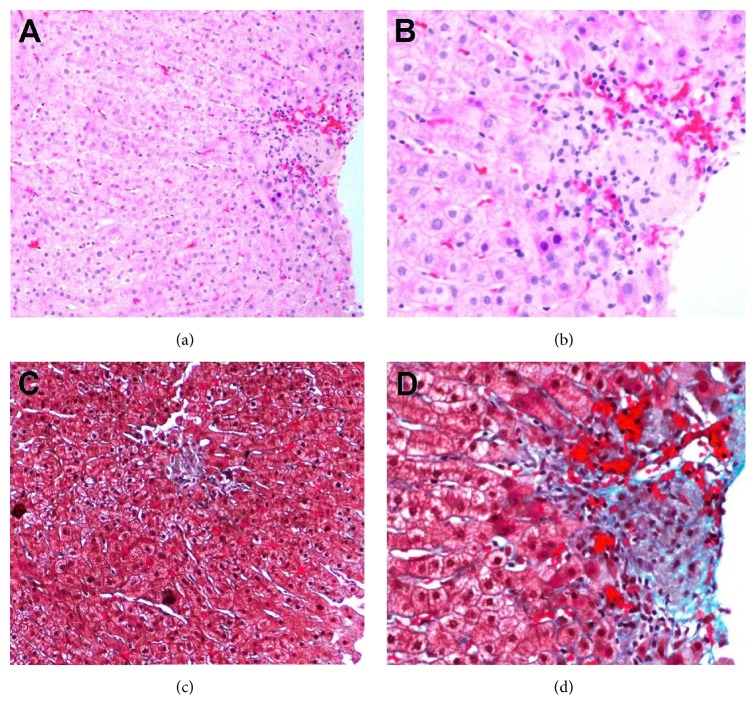
The portal tracts either contain scattered lymphocytes or are entirely devoid of inflammation. In the perivenular areas, Kupffer cells containing ceroid pigments indicative of prior cellular injury and cell turnover are noted. Scattered lymphocytes are also seen in the perivenular zone. Small aggregates of histocytes, “microgranulomata,” are identified in the hepatic lobule and in the perivenular region. (a) and (b) 10x and 20x H&E stain; (c) and (d) 10x and 20x trichrome stain.

On followup, liver enzymes were gradually increasing. On October 2010, ALT and AST were 186 and 260, respectively, while ALP and GGT were 132 and 55, respectively. Subsequently, a liver biopsy was performed and showed a mild nonspecific portal lymphocytic inflammatory infiltrate and aggregates of Kupffer cells containing ceroid pigments in the hepatic lobules and perivenular areas. There were no plasma cells, interface hepatitis, or hepatic rosettes to suggest autoimmune hepatitis. Interestingly, granulomas were identified in the hepatic lobules and in the perivenular regions (Figure 2). A repeat workup for viral and autoimmune etiologies was negative.

On February 2011, her liver enzymes became significantly elevated (ALT 667, AST 665, ALP 221, and GGT 308) and the liver function started to decline (bilirubin 46, INR 1.4, and albumin 31). Based on the possible immune pathophysiology of GA and the presence of liver granulomas, a presumptive diagnosis of a similar immune mediated process involving the liver was made. Treatment with prednisone was initiated at 40 mg daily. The repeat blood work showed a rapid improvement of the liver enzymes and function. Interestingly, an improvement of the skin manifestations was also noted. A repeat liver biopsy was done three months after initiating prednisone. It showed a minor degree of nonspecific portal hepatitis with no fibrosis or granulomas. No features of autoimmune hepatitis were identified. The liver enzymes normalized four months after starting prednisone, which was subsequently tapered and discontinued while azathioprine was initiated. The liver enzymes and function tests continued to be normal on followup.

## 3. Discussion

We reported a unique case of a patient with GA presenting with elevated liver enzymes and findings of hepatic granulomas on pathology. No underlying etiology for the hepatitis was detected despite an extensive workup including two liver biopsies. Based on the possible autoimmune pathogenesis of GA and the fact that corticosteroids are considered one of the treatment options for GA, the patient was treated with prednisone followed by azathioprine which resulted in a rapid and sustained improvement of liver enzymes. Interestingly, an improvement of the skin lesions was also noted which supports the suspicion of a shared autoimmune phenomenon for both conditions. One may argue that the patient may have autoimmune hepatitis. However, the patient did not have any of the characteristic laboratory or histological findings [4]. An atypical presentation of autoimmune hepatitis remains an unlikely possibility. While there are many possible etiologies for liver granulomas such as sarcoidosis, there was no clinical or laboratory suggestion of an alternative etiology in our patient.

GA is rarely reported in association with liver diseases. We performed a literature search in Medline using the search terms “granuloma annulare” in combination with “liver” or “hepatitis.” There were no limitations on language or date of publication. We also manually searched the reference lists for the relevant articles. A total of 10 articles were identified, consisting of case reports with one article describing 2 cases. A summary of the findings is shown in Table 1.

GA has been reported in association with hepatitis C [5], hepatitis B [6, 7], and primary biliary cholangitis [8]. In all cases, the diagnosis of liver disease preceded the onset of GA. In one of the two cases in which GA was associated with hepatitis B, HBV DNA was identified in the skin biopsies but the serum HBV DNA was below the detection range (≤10^3^ copies/mL). This patient received treatment with interferon alpha, which resulted in regression of the skin lesions [7]. GA has been also noted to regress following hepatitis C treatment [5]. In contrast, pegylated interferon treatment for chronic hepatitis C was reported in two cases to trigger GA that resolved following end of therapy [9, 10], which suggests an immunogenic mechanism of GA. Hepatitis B vaccination has been associated with new onset GA in two cases [11, 12]. GA developed in sun exposed areas in two liver transplant recipients, 4 and 18 months after liver transplantation [13]. Given the rare coexistence of GA and liver conditions, it is difficult to know whether there is a true association or just coincidence.

Although GA is usually a benign idiopathic condition, it has been associated with different malignant tumors. We found only a single case report of GA occurring in a patient with hepatocellular carcinoma [14]. GA was diagnosed simultaneously with hepatocellular carcinoma. It was impossible to know the nature of the association as the patient died before receiving treatment for the liver cancer. The occurrence of GA as a paraneoplastic syndrome has been reported with other cancers [15].

We presented a unique case in which liver granuloma were identified in a patient who has GA. A similar association has not been previously reported. Multiple disorders have been associated with liver granulomas [16, 17]. However, a definitive etiology cannot be found in a significant proportion of cases. Interestingly, no granulomas were identified in the second liver biopsy. This may indicate a response to treatment or a patchy involvement resulting in a sampling error. Alternatively, the presence of liver granulomas in a liver biopsy can be a spurious finding and may not necessarily indicate the presence of an underlying granulomatous liver disease [16].

Sarcoidosis, which may mimic GA in its cutaneous form, is a common cause of liver granulomas and has been described in association with GA [18]. While the histopathology findings in our case are not specific, we do not believe that our patient has hepatic sarcoidosis although it is a remote possibility. Hepatic involvement with sarcoidosis is typically associated with a cholestatic rather than a hepatocellular pattern, particularly with the high level of transaminases seen in our case [16]. The patient had no systemic manifestations that suggest sarcoidosis although hepatic sarcoidosis rarely occurs in isolation. The patient had two skin biopsies that were not suggestive of sarcoidosis. While the elevation of serum angiotensin converting enzyme level is a not a very sensitive test for sarcoidosis, it was low in our patient. Similarly, the patient did not have fever, elevated erythrocyte sedimentation rate, or other features suggestive of idiopathic granulomatous hepatitis, which is also characterized by a cholestatic rather than a hepatocellular pattern.

In conclusion, the association between granuloma annulare and liver diseases is rare. We presented a rare case of chronic hepatitis with liver granulomas in association with GA. The patient responded well to prednisone followed by azathioprine treatment. When evaluating a patient with a liver disease, a thorough history and physical examination is essential to asses for potential association with conditions that may have a shared or similar pathophysiology.

## Figures and Tables

**Table 1:** Summary of the reported association between liver disease and granuloma annulare.

First author	Year of publication	Age	Sex	Association
Koizumi	1990	56	Female	Primary biliary cirrhosis
Wolf	1998	51	Female	Hepatitis B vaccine
Granel	2000	65	Female	Hepatitis C
Derancourt	2000	65	Male	Liver transplant
Derancourt	2000	59	Male	Liver transplant
Criado	2004	58	Female	Hepatitis B vaccine
Kluger	2006	52	Female	Hepatitis C treatment
Ma	2006	63	Female	Hepatitis B
Askin	2009	60	Female	Hepatitis B
Ahmad	2013	49	Male	Hepatitis C treatment
Mestre	2014	68	Male	Hepatocellular carcinoma
